# Design of Antimicrobial Peptides with Cell-Selective Activity and Membrane-Acting Mechanism against Drug-Resistant Bacteria

**DOI:** 10.3390/antibiotics11111619

**Published:** 2022-11-13

**Authors:** Seong-Cheol Park, Hyosuk Son, Young-Min Kim, Jong-Kook Lee, Soyoung Park, Hye Song Lim, Jung Ro Lee, Mi-Kyeong Jang

**Affiliations:** 1 Department of Chemical Engineering, Sunchon National University, Suncheon 57922, Republic of Korea; 2 Department of Exhibition and Education, National Marine Biodiversity Institute of Korea, Seocheon 33662, Republic of Korea; 3 LMO Team, National Institute of Ecology (NIE), Seocheon 33657, Republic of Korea

**Keywords:** antibacterial activity, chimeric peptide, cytotoxicity, membrane-acting mechanism

## Abstract

Antimicrobial peptides (AMPs) can combat drug-resistant bacteria with their unique membrane-disruptive mechanisms. This study aimed to investigate the antibacterial effects of several membrane-acting peptides with amphipathic structures and positional alterations of two tryptophan residues. The synthetic peptides exhibited potent antibacterial activities in a length-dependent manner against various pathogenic drug-resistant and susceptible bacteria. In particular, the location of tryptophan near the N-terminus of AMPs simultaneously increases their antibacterial activity and toxicity. Furthermore, the growth inhibition mechanisms of these newly designed peptides involve cell penetration and destabilization of the cell membrane. These findings provide new insights into the design of peptides as antimicrobial agents and suggest that these peptides can be used as substitutes for conventional antibiotics.

## 1. Introduction

Overuse and improper application of antibiotics in the biomedical field has led to the emergence of multidrug-resistant bacteria [1,2,3], making it critical to develop a new generation of antimicrobial agents to replace those that are no longer effective. Several studies have been conducted to identify novel antibiotics from various natural sources [4,5,6]. Antimicrobial peptides (AMPs) are promising such candidates, being host defense molecules that [7,8,9,10,11,12] serve as the initial line of immunological protection against microbial infection in several animals. Although the secondary structures of AMPs are classified into α-helices, β-sheets, extended, and flexible loop structures [13,14], they have common features such as a broad antibacterial spectrum, amphipathic properties, short amino acid length, cationic net charge, and rapid killing kinetics [7,8,9,10,11,12]. Furthermore, the low levels of resistance-inducing behavior of AMPs make them superior to small-molecule antibiotics [12]. Membranolytic AMPs usually cause potential membrane changes or direct membrane disruption via four distinct mechanisms: (1) barrel stave, (2) carpet, (3) toroidal, and (4) aggregation [15,16,17,18,19,20]. Other AMPs enter the cytoplasm of microbial cells via spontaneous lipid-assisted and receptor- or channel-mediated translocation. Several mechanisms including transient or disordered pore formation and lipid phase alteration enable AMP penetration [21,22]. However, the AMP uptake mechanisms of many bacteria-penetrating peptides and their cytoplasmic targets have not yet been elucidated.

Previous studies have reported that amphipathic α-helix structures with hydrophobic and hydrophilic residues on opposite sides of a cationic α-helix in AMPs have effective antibacterial properties [23,24]. Based on the amino acid combination, length, charge, and hydrophobicity of synthetic AMPs, they can be specifically designed to interact with specific regions of the microbial cell membrane [25,26]. Tryptophan (Trp, W) strongly interacts with zwitterionic liposomes that resemble cell membranes in a hydrophobic manner, and the highly hydrophobic aromatic ring of Trp is preferentially buried in the hydrophobic portion of the lipid bilayer. Additionally, negatively charged phosphate groups in microbial membranes prefer the polar regions of positively charged lysine (Lys, K) and arginine (Arg, R) residues. However, several AMPs are cytotoxic at higher doses and unsuitable for use in vivo, thus numerous studies have investigated novel peptides with antibacterial activity that do not display cytotoxicity at high concentrations [26,27]. 

In this study, we designed and synthesized a set of α-helical peptide analogs by substituting amino acids, particularly tryptophan, to compare the effects of differences in peptide length and amino acid position on antibacterial activity. The antibacterial efficacy of the synthesized peptides was evaluated against various drug-susceptible and drug-resistant bacterial strains. The hemolytic and cytotoxic effects of the peptides on rat red blood cells (rRBCs) and HaCaT (human keratinocyte) cells were investigated. Finally, the mechanism of action of the peptide variants was examined in several liposomes, that acted as artificial cell membranes. Our findings provide evidence that the designed peptides can be used as antimicrobial agents for several natural infections without cytotoxic effects.

## 2. Results and Discussion

### 2.1. Designing and Modeling of α-Helical Peptides

Antimicrobial peptides (AMPs) act via intracellular and cell membrane modes of actions. The intracellularly mediated microbial killing and growth inhibition are due to flocculation of intracellular contents [28], alteration of cytoplasmic membrane septum formation [29], inhibition of cell wall [30], nucleic acid [31] and protein synthesis [32], and inhibition of enzymatic activity [33]. Unlike the mechanism of action of conventional antibiotics, the membrane-permeabilizing actions of AMPs, such as the ‘toroidal pore model’ [34,35], ‘barrel-stave model’ [34,35], and ‘carpet model’ [34,36], are considered as ways to overcome drug-resistance. Moreover, the selectivity of AMPs for bacterial membrane permeabilization, compared to host–cell membrane permeabilization, depends on cell membrane components. Therefore, the peptides used in this study were designed to facilitate the attachment and transmembrane action specifically on bacterial membranes. Previous studies on the adaptation of membrane proteins to the membrane–water interface provide useful information for designing membrane-acting peptides [37,38,39,40]. The interaction of amino acid residues with the membrane–water interface can be important for the binding, translocation, and function of AMPs. Thus, we used lysine (Lys), arginine (Arg), tryptophan (Trp), isoleucine (Ile), and glutamic acid (Glu) in our model peptides. Since the bacterial membrane is negatively charged, positively charged amino acids in the AMP sequence are required for attachment to the bacterial surface. Lys and Arg enable binding to the bacterial surface via electrostatic interactions, and their long and flexible alkyl chains are widely located in the hydrophobic core of the membrane. Glu was used to change the cation strength and induce an electrostatic force between peptides. In contrast, the Ile and Trp residues were used to complete the amphiphilic helical structure. In particular, the Trp side chain, with its indole NH, is close to the lipid carbonyl ester and is anchored rather rigidly to the interface. Therefore, the position of tryptophan in the peptide sequence may cause a change in the lipid orientation. Thus, we designed antimicrobial peptide analogs considering three issues: (1) the positions of Trp, (2) changes in cation strength by Lys, Arg, or Glu, and (3) the length of the α-helical structure. These peptides were designed by positional alterations of two tryptophan residues, substitution between lysine and arginine residues, net charges, and peptide lengths (10-mer and 14-mer).

**Figure 1:**
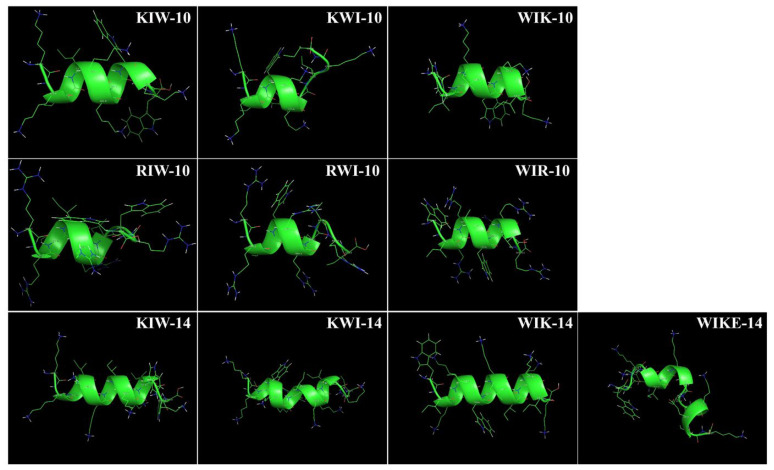
PEP-FOLD model of KXW peptides. Structures of designed peptides were modeled using PEP_FOLD server (http://mobyle.rpbs.univ-paris-diderot.fr/cgi-bin/portal.py#forms::PEP-FOLD).

The sequences of the 10 designed peptides are listed in Table 1. All peptides were expected to form a helical structure in partial or full sequence by using PEP_FOLD server (Figure 1). Positioning the two tryptophan residues in the middle of the peptide sequence partially unraveled or loosened the helical structure (Figure 1). The number of helical turns increased with sequence length; however, the WIKE-14 peptide in which Glu was substituted with Lys was predicted to form a helix-to-helix structure. 

### 2.2. Cell-Selective Effects of Designed Peptides against Bacterial Strains and Mammalian Cells

The synthesized peptides were confirmed by RP-HPLC using C18 columns, and the molecular mass of each peptide was determined by MALDI-TOF/MS. We then investigated the antibacterial activities of the ten peptide variants against five pathogenic bacteria and compared them with those of melittin [41], an established antimicrobial agent (Table 1). Among the peptides with 10 amino acids, the MIC values of peptides with Arg residues were lower than those of peptides with Lys residues. Peptides with 14 amino acids showed better antibacterial activity than peptides with 10 amino acids. Among all peptides, those with Trp at the N-terminus (WIK-10, WIR-10, and WIK-14) had more potent antibacterial effects than the others. WIKE-14 had a higher MIC value than the other peptides. This indicates that the peptide binding affinity to the bacterial membrane is weakened due to lower cationic charge.

**Figure 2:**
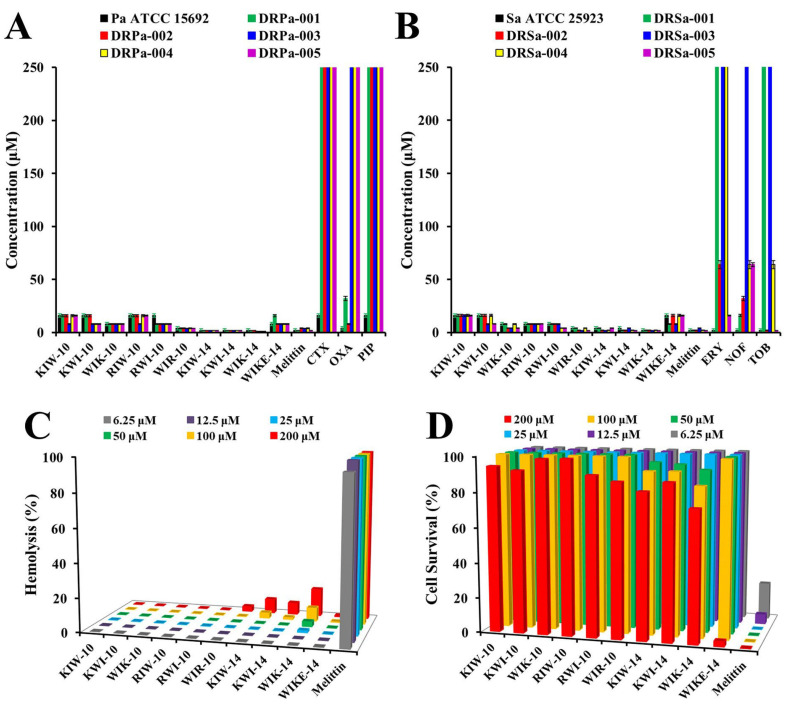
Antibacterial (**A**,**B**), hemolytic (**C**), and cytotoxic (**D**) effects of synthetic peptides. (**A**,**B**) MICs of peptides were evaluated against drug-susceptible bacteria (*P. aeruginosa* ATCC145692 and *S. aureus* ATCC25923) and drug-resistant bacteria (DRPa-001~005 and DRSa-001~005), compared to cefotaxime (CTX), oxacillin (OXA), piperacillin (PIP), erythromycin (ERY), norfloxacin (NOF), and tobramycin (TOB). (**C**) Hemolysis and cytotoxicity (**D**) of peptides was investigated in rat erythrocytes and HaCaT cells, respectively.

The antibacterial activity of the peptides was tested against 10 drug-resistant *P. aeruginosa* (DRPa) and *S. aureus* (DRSa) strains that were clinically isolated from patients and compared to conventional antibiotics (Figure 2A,B). Notably, three 14-mer peptides, KIW-14, KWI-14, and WIK-14, had increased potency against drug-resistant bacteria than six 10-mer peptide variants. In contrast, the MICs of all peptides were remarkably lower than those of the conventional antibiotics. In addition, peptides with Trp residues in the N-terminal region were more active than the others against drug-susceptible and drug-resistant bacteria.

AMPs must be nontoxic to be developed as effective antimicrobial therapeutics. Therefore, we evaluated the hemolytic and cytotoxic effects of all synthetic peptide variants on rat red blood cells (RBCs) and HaCaT cells. As shown in Figure 2C, melittin had a strong hemolytic effect even at a low concentration of 6.25 µM, whereas all 10 peptides showed low hemolysis even at a high concentration of 200 µM. On the other hand, in HaCaT cells, 200 µM melittin resulted in less than 20% cell survival, but the cells treated with the same concentration of the peptides survived, except for WIKE-14 (Figure 2D). These findings suggest that the peptides exhibit selectivity between bacterial and human cells.

**Figure 3:**
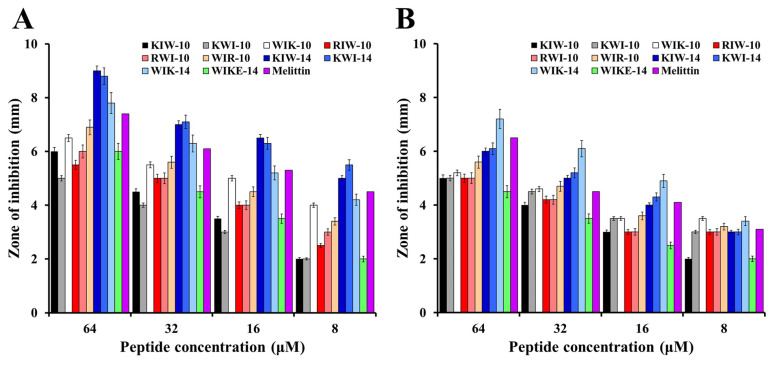
Antibacterial activity of peptides against *E. coli* (**A**) and *S. aureus* (**B**) determined with the agar-diffusion assay.

To investigate the relationship between the antibacterial activity and permeability of the peptides, an inhibition zone assay was performed (Figure 3). After adding 10 µL of the peptide solutions at the indicated concentrations to the punched wells, clear zones corresponding to the inhibitory effect of each peptide against *E. coli* and *S. aureus* were determined. As shown in Figure 3, KIW-14, KWI-14, and WIK-14 had increased inhibitory activity than other peptides and the size of the clear zone increased in a peptide dose-dependent manner. These results were almost similar to the patterns of antimicrobial activity in the broth medium. Antimicrobial tests on agar plates can be used to understand the effect of viscosity on peptide activity and to compare the diffusion capacity of peptides. We propose that 14-mer peptides can rapidly inhibit the growth of surrounding bacteria due to their excellent diffusion ability. We expect that this ability can be applied in clinics for gel-type antibacterial pads or wound-healing bands.

### 2.3. Mode of Action of Peptides

**Figure 4:**
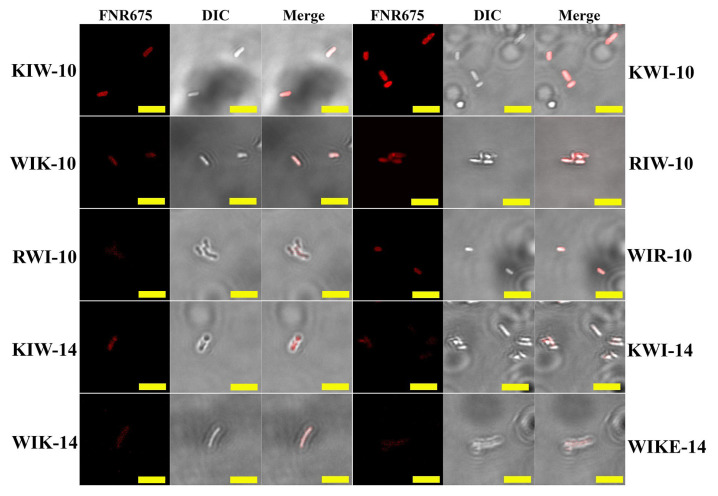
Cellular distributions of peptides in *E. coli* cells. *E. coli* cells incubated with Flamma675-labeld peptides at each MIC for 30 min were observed by Confocal Laser-Scanning Microscopy (CLSM). Bar = 5 µm.

To investigate the cellular distribution of the peptides in bacterial cells, *E. coli* cells were incubated with Flamma675-labeled peptides (BioActs, Incheon, Korea), and fluorescence was observed using confocal laser scanning microscopy (CLSM). As shown in Figure 4, all Flamma675-labeled peptides accumulated on *E. coli* surfaces, suggesting that the growth-inhibitory effect of the synthesized peptides against bacterial cells may result from interactions between the peptide analogs and the bacterial membrane.

**Figure 5:**
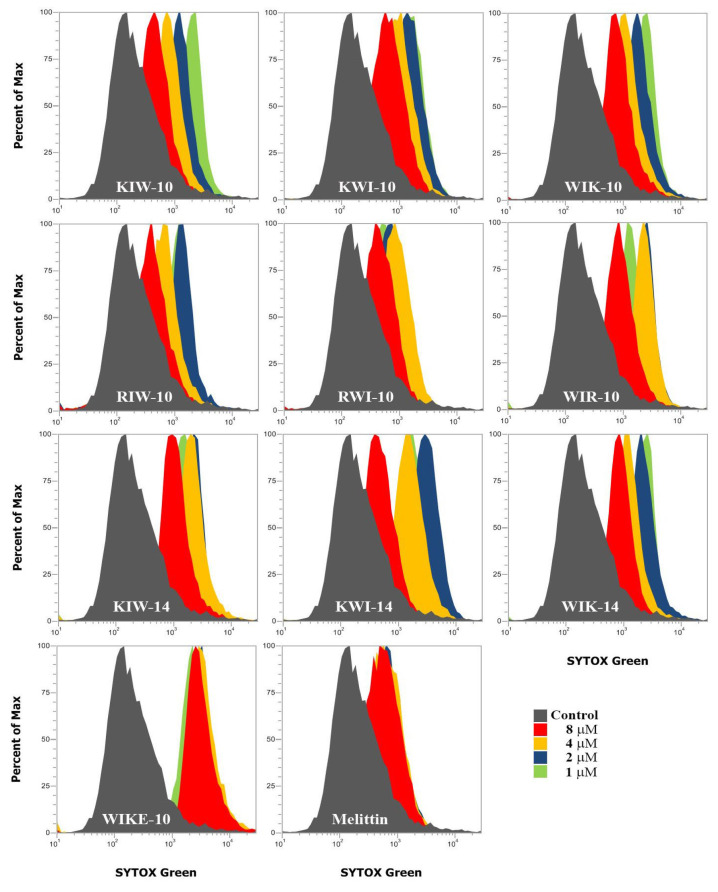
SYTOX Green uptake of peptides in *E. coli* cells. After adding peptides in SYTOX Green pre-incubated *E. coli* cells at indicated concentrations, *E. coli* cell fluorescence was measured using flow cytometry.

Furthermore, to determine whether the peptides affected the permeability of the bacterial membrane, we performed a SYTOX Green uptake assay. SYTOX Green is an impermeable probe that enters the cell when the integrity of the cell membrane is changed or disrupted, resulting in binding to nucleic acids and emitting green fluorescence. Peptides at 1, 2, 4, and 8 µM concentrations were used to treat *E. coli* stabilized by pretreatment with SYTOX Green. Analysis using flow cytometry revealed that all peptides permeabilized the *E. coli* cell membrane and allowed the probe to penetrate, even at the lowest concentration of 1 µM (Figure 5). 

**Figure 6:**
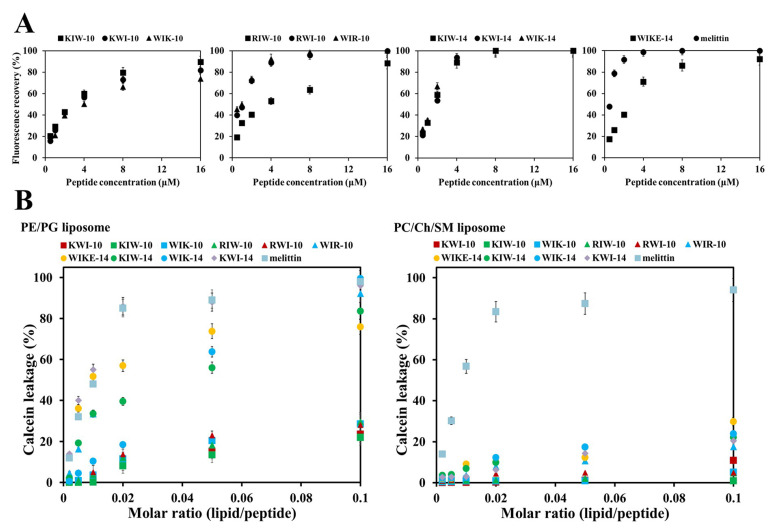
Peptide-induced depolarization of bacterial membranes (**A**) and calcein leakage from artificial liposomes (**B**). (**A**) The indicated peptides were added to intact *E. coli* ATCC 25922 that had been pre-equilibrated for 60 min with diS-C3-5. Fluorescence recovery was measured for 60 min (at 5 min intervals) after the peptides had been mixed with the bacteria. (**B**) Calcein leakage from calcein-entrapped small unilamella vesicles (SUVs) composed of PE/PG (7:3, *w*/*w*, bacterial membrane) and PC/Ch/SM (1:1:1, *w*/*w*/*w*, mammalian plasma membrane).

To further investigate the bacterial growth inhibitory effects of the synthesized peptides, we used the membrane potential-sensitive fluorescent probe DiSC3-5 to assess the ability of the peptides to induce membrane depolarization in antibiotic-susceptible *E. coli* (ATCC 25922). The peptide-induced changes in membrane permeability leading to dissipation of the transmembrane potential were monitored by measuring the increase in fluorescent emission caused by the release of the membrane potential-sensitive dye, DiSC3-5. As shown in Figure 6A, the magnitude of the bacterial membrane depolarization by the peptides and melittin varied significantly, whereas the maximum depolarization of *E. coli* treated with 10-mer peptides was detected at a peptide concentration of 16 µM. The depolarization of 14-mer peptide treated cells, except WIKE-14, was detected at about 4 µM, the MIC for *E. coli* strains. The dose-dependent dissipation of the bacterial membrane potential by the peptides indicated a correlation between the antibacterial activity and membrane potential disruption. These results suggest that 14 amino acid peptides were more potent in inducing membrane depolarization in intact *E. coli* than the 10 amino acid peptide analogs.

It is not possible to selectively elucidate whether peptides act only on the cell membrane in live bacterial cells. Therefore, we prepared liposomes with or without calcein to investigate the action of the peptides in artificial membranes. Peptide binding and partitioning into lipid bilayers were examined by recording tryptophan (Trp, W) fluorescence emission spectra in the presence of vesicles composed of phosphatidylethanolamine (PE), phosphatidylglycerol (PG) (7:3, *w*/*w*), phosphatidylglycerol (PG):cardiolipin (CL) (1:1, *w*/*w*), and phosphatidylcholine (PC):cholesterol (CH):sphingomyelin (SM) (1:1:1, *w*/*w/w*) (Table 2). The use of two buffers is to understand the antimicrobial activity in low ionic strength (buffer I, topical treatments) and high ionic strength (buffer II, physiological environment of human fluids) conditions. In aqueous conditions without any liposomes, the maximum emission intensity of all antibacterial peptides was observed at 352–357 nm in buffer I and II. However, in the presence of PE:PG vesicles (bacterial membrane-mimicking environments), the maximum Trp emission intensity was detected at 318–343 nm in buffer I, suggesting that all antibacterial peptides strongly interacted with the bacterial membrane, and the interaction shifted the Trp intensity of the peptides. The maximum emission intensity of all antibacterial peptides at 318–346 nm in buffer II reflects their binding to the bacterial membrane. In the presence of PG:CL (1:1, *w*/*w*) vesicles (inner membrane mimicking environments of Gram-negative bacteria), the results were similar to those in the PE:PG condition. On the other hand, in PC:CH:SM (1:1:1, *w*/*w/w*) liposomes (mammalian membrane-mimicking environments), the maximum Trp emission of the designed peptides shifted to 0–6 nm compared with the aqueous condition, indicating no interaction with PC:CH:SM liposomes. These results were consistent with the lack of cytotoxic effects of the synthetic peptides (Figure 2C,D).

To further investigate the effect of the peptides on cell membranes, the membrane-permeabilizing abilities of the designed peptides were measured by calcein leakage from various cell membrane-mimicking liposomes, which comprised PE:PG (7:3, *w*/*w*) as the bacterial membrane and PC:CH:SM (1:1:1, *w*/*w*/*w*) as the mammalian plasma membrane analogs. The dye leakage value indicates the ability of an antimicrobial agent to disrupt the membranes. As shown in Figure 6B, bacterial liposomes showed a significant dose-dependent release of calcein in the presence of melittin or 14-mer peptides, such as KIW-14, KWI-14, WIK-14, and WIKE-14. The 10-mer peptides either showed no significant release of calcein (KIW-10, KWI-10, and WIK-10) or a weak leakage (RIW-10, RWI-10, and WIR-10) (Figure 6B). These results suggest that the 14-mer peptides directly disrupt the bacterial membrane, and the 10-mer peptides only weakly damage the membrane during bacterial membrane penetration. However, none of the peptides exhibited remarkable calcein release from the mammalian plasma membrane, whereas melittin elicited significant membrane disruption even at low concentrations (Figure 6B). These results are consistent with the observed antibacterial and cytotoxic effects. 

**Figure 7:**
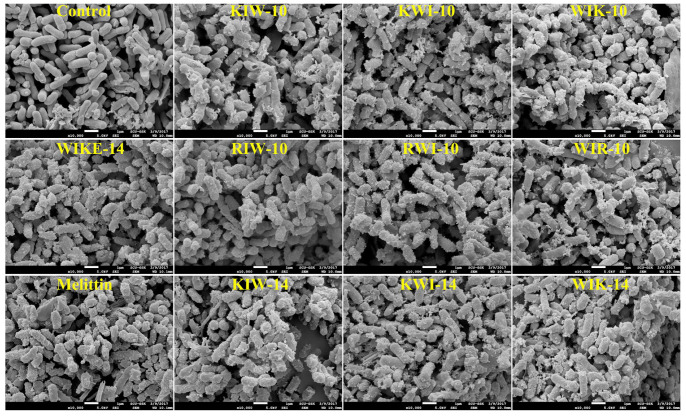
Peptide-induced morphological changes in *E. coli* cells. All peptides were treated at minimum inhibitory concentrations (MICs).

Morphological changes on the surface of *E. coli* cells incubated with the indicated peptides were observed using scanning electron microscopy (SEM). The peptide-untreated cells exhibited a smooth surface without cell debris or any changes (Figure 7, control). However, the peptide-treated cells were wounded and shrunken with small blebs, indicating damage to cell membranes by the peptides (Figure 7). Thus, our findings suggest that the designed peptides exhibit potent antibacterial activity by destabilizing the cell membrane or by membranolysis.

### 2.4. In Vivo Antibacterial Effects of the Selected Peptides

**Figure 8:**
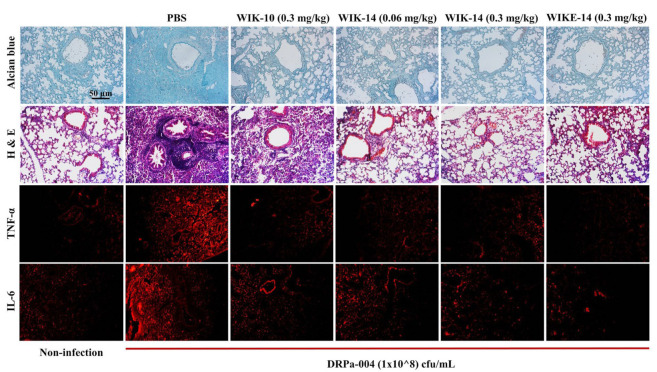
*In vivo* antibacterial effects of WIK-10, WIK-14, and WIKE-14 peptides. After 1 h treatment with DRPa (1 × 10^8^ cfu/mL) by intratracheal (i.t.) administration, Balb/c mice that were treated with PBS and the indicated peptides (n = 4) by i.t. administration were euthanized after three days. Inflammatory effects of the peptides were examined by immunohistology using lung tissues of *P. aeruginosa*-induced pneumonia mice. TNF-α and IL-6 were detected using monoclonal PE-label antibody.

*Pseudomonas aeruginosa* is the most commonly isolated microorganism in patients with nosocomial pneumonia. To elucidate the *in vivo* antibacterial effects of WIK-10, WIK-14, and WIKE-14 peptides against drug-resistant *P. aeruginosa* infection, we administered high concentrations of the bacteria by intratracheal (i.t.) intubation in an acute pneumonitis model. Peptides were administered i.t. to enable rapid action without interference from proteolytic enzymes. After bacterial infection, all mice in the PBS-treated group died within 3 days. Although the mice in the peptide-treated group survived for a long time, the endpoint was set at 3 days. To analyze the severity and progression of chronic pulmonary disease, Alcian blue staining, H&E staining, and immunostaining were performed using paraffin sections from the excised tissue. Alcian blue is used to visualize acidic epithelial and connective tissue mucins. As shown in Figure 8, bacteria-infected tissues had apparent mucus deposition, which was reduced in tissues with peptide treatment, similar to uninfected tissues. H&E staining (Figure 8) revealed inflammatory cell infiltrates around the bronchus and alveoli in the infected mice, indicating severe infection. WIK-10 (0.3 mg/kg) treatment showed infiltration only in the alveoli, indicating moderate infection. Mice treated with other peptides displayed substantially lower levels of inflammatory infiltrates, similar to the lung tissues of non-infected mice (WIK-14 at 0.06 mg/kg and WIKE-14 at 0.3 mg/kg had mild symptoms, and WIK-14 at 0.3 mg/kg had no pathological symptoms).

To investigate the expression of the proinflammatory cytokines, interleukin (IL)-6 and tumor necrosis factor α (TNF-α), PE-labeled cytokine antibodies were used in each tissue. Mice treated with the peptides had greater inhibition of cytokine secretion than those treated with PBS. Interestingly, mice treated with WIKE-14 showed slightly more inflammatory cell infiltrates than those treated with WIK-14, but secreted fewer cytokines.

**Figure 9:**
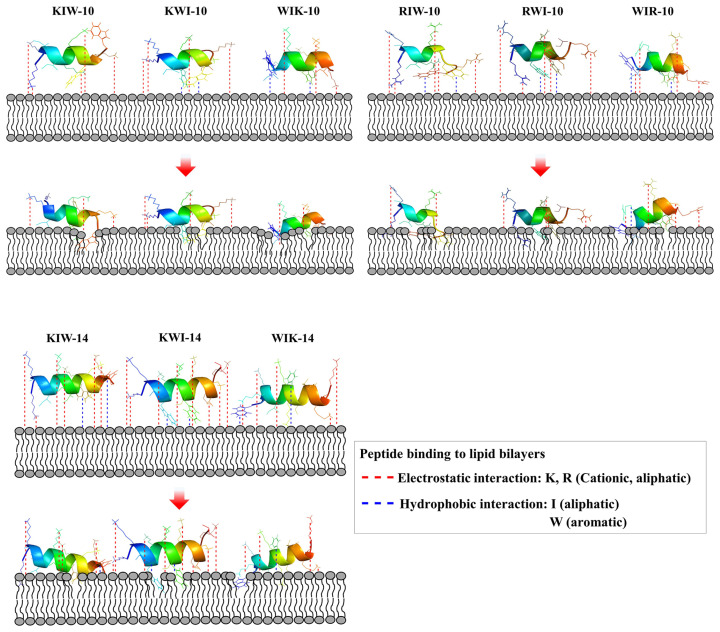
Schematic mode of action of the designed peptides in bacterial membranes. Red-dotted lines indicate electrostatic interaction between cationic amino acids and anionic head group of lipids. Hydrophobic interaction between hydrophobic amino acids and fatty acids of lipids.

Lipopolysaccharides in the outer membrane of Gram-negative bacteria and teichoic acid in the peptidoglycan layer of Gram-positive bacteria contain phosphate, indicating that all bacteria have a negatively charged surface. Positively charged amino acids, such as lysine and arginine, in AMPs facilitate attractive binding to bacteria via electrostatic interactions. Membrane-acting AMPs must pass through the outer membrane or the peptidoglycan layer into the cytoplasmic membrane, where they exhibit membranolytic action. Figure 9 shows the expected actions of the peptides on the cytoplasmic membrane. The amphipathic helical structure is induced by the attachment of Lys or Arg to the membrane surface. Because lysine and arginine have long aliphatic parts, they tend to be located in the hydrophobic part of the bilayer. Although the Trp residues in our designed peptides were hydrophobic, the amide group of the fused aromatic ring was preferentially located in a polar environment. However, since Ile has an aliphatic chain, it has the property of making hydrophobic bonds with lipid fatty acids, resulting in insertion into the membrane. All peptides act similarly on the cytoplasmic membrane, but the range of their action on the membrane is widened based on the position of the Trp residues and the distance between Trp residues. We propose that the superior antibacterial activity of WIK-10, WIR-10, and WIK-14 is due to the long distance between the Trp residues, which causes significant cell membrane damage.

## 3. Materials and Methods

### 3.1. Materials

Calcein, cholesterol (Ch, from ovine), cefotaxime, erythromycin, norfloxacin, oxacillin, piperacillin, and tobramycin were obtained from Sigma-Aldrich Co. (St. Louis, MO, USA). Additionally, 9-fluorenylmethoxycarbonyl (Fmoc)-amino acids and ethyl 2-cyano-2-(hydroxyimino)acetate (Oxyma Pure) were bought from CEM Co (Matthews, NC, USA). Cardiolipin (CL, from *Escherichia coli*), phosphatidylcholine (PC, from egg), phosphatidylethanolamine (PE, from *E. coli*), phosphatidylglycerol (PG, from *E. coli*), and sphingomyelin (SM, from egg) were from Avanti Polar Lipids (Alabaster, AL, USA). SYTOX Green was purchased from Molecular Probes (Eugene, OR, USA). All other materials were analytical reagents.

### 3.2. Solid-Phase Peptide Synthesis by Microwave

Peptides were synthesized by a Liberty Microwave Peptide synthesizer (CEM Co.). To generate amidated peptides at carboxyl-termini, Rink Amide ProTide Resin (0.58 mmol/g) was used. Fmoc deprotection was completed using 20% piperidine in N,N-Dimethylformamide (DMF). Coupling of each Fmoc-amino acid was achieved in the presence of diisopropyl carbodiimide and Oxyma in DMF. To generate N-terminal fluorescently labeled peptides, Flamma675-carboxylic acid was added to the peptide-bound resin. The protecting groups and peptides on the resin were removed using trifluoroacetic acid(TFA)/diH_2_O/triisopropylsilane (95: 2.5: 2.5, *v*/*v*/*v*) for 40 min at 40 °C. The filtrated peptide solution was precipitated and washed in ice-cold diethyl ether. The solid powder was isolated by centrifugation and then dried under a vacuum. The purification of crude peptides was done on a C18 column (Zorbax, 21.2 × 250 mm, 300Å, 7-μm) on a Shimadzu semi-preparative HPLC system, using a 10–90% acetonitrile gradient in water with 0.05% TFA for 80 min. The purity and molecular masses of the isolated peptide were measured on an analytical HPLC system and a matrix-assisted laser desorption ionization (MALDI) mass spectrometer (Kratos Analytical Ins.), respectively.

### 3.3. Antibacterial Assay by a Microdilution Method

*Pseudomonas aeruginosa* (ATCC 15692), *Escherichia coli* (ATCC 25922), and *Staphylococcus aureus* (ATCC 25923) were purchased from the American Type Culture Collection. *Listeria monocytogens* (KCTC 3710) and *Bacillus subtilis* (KCTC 1998) were from the Korean Collection for Type Cultures. Drug-resistant *S. aureus* (DRSa-001~005) and *P. aeruginosa* (DRPa-001~005) were collected from Paik Hospital of Inje University in Busan, South Korea. Pre-cultured bacteria grown to mid-log phase were suspended in 10 mM sodium phosphate (SP, pH 7.2) or phosphate-buffered saline (PBS, pH 7.2) with 10% culture media. Bacterial suspensions were added to each peptide dilute (final 5 × 10^5^ colony-forming units (cfu)/mL), after which the plates were incubated at 37 °C for 24 h. The turbidity of each well was measured at the absorbance at 600 nm and the morphology of bacterial growth was observed under light microscopy. MICs (Minimum inhibitory concentrations) were defined as the lowest concentration of peptide that expresses the complete inhibition of bacterial growth [42,43].

### 3.4. Hemolysis and Cytotoxicity

Fresh rat erythrocytes were collected and washed by centrifugation at 1000× *g* for 10 min with PBS. The rat RBCs (8% (*v*/*v*)) were added in a two-fold serially diluted peptide with PBS, followed by incubation for 1 h at 37 °C. After centrifugation of samples at 1000× *g* for 10 min, the supernatants were collected and their absorbance was measured at 414 nm. The 8% RBCs dissolved in PBS and RBCs treated with Triton X-100 were considered as the negative and positive controls, respectively. The hemolysis activity was calculated using the formula, % hemolysis = [(Abssample − Absnegative)/(Abspositive − Absnegative)] × 100 [43,44].

To examine the cytotoxic effect of peptides, HaCaT (human keratinocyte) cells were sub-cultured in Dulbecco’s modified Eagle medium (DMEM) containing 10% fetal bovine serum, 100 µg/mL streptomycin, and 100 U/mL penicillin at 37 °C in a 5% CO_2_ chamber. The cells seeded into 4 × 10^3^ cells/well were incubated for 24 h. Peptide solutions diluted with DMEM were incubated with cells for 24 h at 37 °C. 3-(4,5-Dimethyl-2-thiazolyl)-2,5-diphenyltetrazolium Bromide (MTT) solution was added to the cells, followed by additional incubation for 4 h. After removing the media, 200 µL of dimethyl sulfoxide was added to the wells. Absorbance of each well was measured at 570 nm using a microtiter reader (Molecular Devices Emax, CA, USA) [44,45].

### 3.5. Inhibition Zone Assay

*E. coli* and *S. aureus* cells were washed with SP buffer. Fifteen milliliters of underlay agarose gel containing 0.03% (*w*/*v*) culture medium, and 1% (*w*/*v*) agarose in the presence of 5 × 10^5^ cfu/mL, was poured into a Petri dish, and the gel was solidified. Peptide solution (8 µL) was added to the punched wells (3 mm diameter) after which the plate was incubated at 37 °C for 3 h. A gel solution containing 6% culture media and 1% agarose was poured on underlay gel, followed by further incubation at 37 °C for 24 h. 

### 3.6. Membrane Depolarization 

The membrane-depolarizing activity of peptides was determined using DiSC3-5, a membrane potential-sensitive dye, on intact *S. aureus* and *E. coli* cells. Pre-grown bacteria were washed three times with 5 mM HEPES buffer containing 20 mM glucose (pH 7.2). The cells were suspended to A_600_ = 0.05 in 5 mM HEPES buffer containing 20 mM glucose and 0.1 M KCl (pH 7.2), and the dye was added to a final concentration of 100 nM and then incubated for about 60 min until a constant fluorescence was achieved. Peptides were then added to bacterial suspensions. The fluorescence was recorded (Ex 622 nm and Em 670 nm) for 30 min [43].

### 3.7. Confocal Laser-Scanning Microscopy (CLSM)

*E. coli* treated with Flamma^®^675-labeled peptides were observed on a CLSM. Cells were inoculated and adjusted as the above procedure of antimicrobial assay. Flamma675-labeled peptides were added to 100 μL of the cell suspensions at each MIC, after which the cells incubated for 30 min were washed by centrifugation at 4000 rpm for 5 min in three times with ice-cold PBS buffer. Localization of Flamma675-labeled peptides was then examined using CLSM (A1R HD 25; Nikon, Tokyo, Japan).

### 3.8. SYTOX Green Uptake Assay

Pre-grown E. coli cells were washed and suspended to 2 × 10^7^ cells/ ml in SP buffer. The cells pre-treated with 1 μM SYTOX green for 15 min in the dark were incubated with the peptides in appropriate concentrations; the bacteria were analyzed by flow cytometry (Attune NxT acoustic focusing cytometer, Thermo Fisher Scientific Co., Waltham, MA, USA) [44].

### 3.9. Small Unilamellar Vesicles Preparation

SUVs were prepared by sonication and the freeze–thaw method, respectively [27,28]. The lipid mixtures in chloroform were dried in a glass tube under nitrogen, after which they were lyophilized overnight. Dry lipid films suspended in 2 mL buffer preheated at 50 °C were vortexed. SUVs were prepared by sonication in a bath-type sonicator for 30 min. After the vesicles were prepared, unilamella vesicles were extruded 15 times through 0.05 µm polycarbonate membranes for SUVs using an Avanti Mini-Extruder (Avanti Polar Lipids inc., Alabaster, AL, USA), to ensure a homogeneous population. Their concentration was determined by a standard phosphate assay [44]. 

### 3.10. Tryptophan Fluorescence Blue Shift

To analyze peptide binding and partitioning on/into the lipid bilayer, the fluorescence emission spectrum of Trp residues was monitored upon the addition of SUVs, composed of PC/CH/SM (1:1:1, *w*/*w/w*) or PE/PG (7:3, *w*/*w*). A 2 µM of peptide solution was added to 1 mL of buffers or vesicles at a total lipid concentration range of 0 to 600 µM. This assay was performed in two buffers: buffer I, 10 mM HEPES containing 10 mM NaCl (pH7.4) and buffer II, 10 mM HEPES containing 150 mM NaCl and 5 mM MgCl_2_ (pH 7.4). Tryptophan fluorescence was excited at 280 nm, and the emission spectrum was measured from 290 to 510 nm in 1 nm increments with 1 sec signal averaging. Background and lipid scattering signals were subtracted. The wavelength of the maximum fluorescence emission was recorded [44]. 

### 3.11. Calcein Leakage Assay

The permeabilization of peptides against artificial liposomes was accessed by measuring calcein leakage. Calcein-entrapped liposomes were prepared by freeze–thaw cycles. In brief, the dried lipid was suspended with dye solution (10 mM HEPES containing 80 mM calcein and 1 mM EDTA, pH 7.4), vortexed for 1 min, and left for 30 min at 50 °C. To make large unilamellar vesicles (LUVs), the suspension was frozen and thawed at 50 °C for nine cycles and extruded 15 times through 0.2 μm polycarbonate filter. Calcein-loaded vesicles were separated by a Sephadex G-50 column in buffer (10 mM HEPES, 1 mM EDTA, 120 mM KCl, pH 7.4). Then, 2.5 μM LUVs were incubated with 250 and 25 nM peptides. The calcein dequenching was time-dependently monitored by a spectrofluorometer (Perkin-Elmer LS55) at Ex 480 nm and Em 520 nm. The 0 and 100% releases were determined using only buffer (F0) and 0.03% (*w*/*v*) of Triton X-100 (Ft), respectively. The percentage of released calcein was calculated in the formula [44,45,46]: Release (%) = 100 × (F − F0)/(Ft − F0)

### 3.12. Scanning Electron Microscopy (SEM) Preparation

Pre-cultivated *E. coli* cells (1 × 10^8^ cells/mL) were incubated with peptides at a MIC value for 2 h. Bacterial cells were attached on glass slides coated by poly-L-Lysine coated and fixed with 100 mM HEPES buffer containing 2% glutaraldehyde (*v*/*v*) (pH 7.4) for 12 h at RT. The PBS-washed cells were subsequently dehydrated in an OTTIX shaper (Diapath S.p.A, Bergamo, Italy), followed by chemical dry with hexamethyldisilazane. Samples were sputter-coated by gold-palladium and observed under field emission SEM (JSM-7100F; JEOL, Ltd., Tokyo, Japan).

### 3.13. Murine Infection Model

All animal experiments and procedures were investigated with the approval of the institutional animal care and use committee (IACUC) in Sunchon national university, South Korea (SCNU IACUC-2019-10). Ten-week-old female Balb/c mice (Koatech Co., Pyongtaek, Gyeonggido, Korea) were anesthetized by inhalation of isoflurane in pure oxygen and instilled intratracheally with 1 × 10^8^ cfu/mL of DRPA-005 in 100 µL of PBS. After one hour of exposure, each peptide was intratracheally administered at the indicated dose in 50 µL of PBS. Mice were monitored for signs of morbidity and euthanized on the third day after bacterial exposure. Mice were euthanized by CO_2_ inhalation, and the infected lung tissues were excised and fixed in 4% paraformaldehyde. The fixed tissues were dehydrated using a series of ethanol solutions (50−100%) and embedded in paraffin. The tissues were sectioned in a thickness of 5 µm (Leica microtome, Deerfield, IL, USA). Histopathology of the lung tissues was performed by Alcian blue and hematoxylin and eosin (H&E) staining. Immunohistology was analyzed by using the monoclonal antibody of PE-conjugated anti-IL-6 and anti-TNF-α (BioLegend, San Diego, CA, USA). The stained tissue sections were observed under a fluorescence microscope (OPTINIT KCS3-160S; Korea Lab Tech).

### 3.14. Statistical Analysis

The mean values of at least four independent determinations ± SD were calculated using Excel software (Student *t*-test).

## 4. Conclusions

In summary, we designed 10 peptide analogs with repositioned Trp based on Arg, Glu, Lys, Ile, and Trp amino acids. Considering the balance between antibacterial activity and cytotoxicity, 10-mer peptides using Lys have less antibacterial activity and cytotoxicity than Arg-containing peptides, and 14-mer peptides showed greater antibacterial activity and cytotoxicity than 10-mer peptides. When Trp was located near the N-terminus, antibacterial activity and toxicity simultaneously increased at a high concentration (200 µM). However, their therapeutic index is higher than that of peptides with other Trp-positioning, indicating that they have better potential for clinical applications. Interestingly, WIKE-14, with reduced cation strength, had lower antibacterial activity than other peptides *in vitro*, but had increased antibacterial activity *in vivo*. In particular, the peptide inhibitory effects on cytokine secretion showed the strongest effects among the tested parameters. All peptides exerted antibacterial activity by destabilizing the cell membrane or by membranolysis. Although further research is required, our findings indicate that at least three helix turns are needed for the clinical application of membrane-active AMPs.

## Figures and Tables

**Table 1:** Sequences and antibacterial activities of the designed peptides.

Name		MIC (μM)					
	Sequence	Molecular Mass (Da)	Ec *	Pa *	Sa *	Bs *	Lm *
KIW-10	KKIIKKIWKW-NH_2_	1369	8(64)	16(64)	16(128)	4(32)	4(128)
KWI-10	KKIWKKWIKI-NH_2_	1369	8(64)	16(64)	16(128)	2(32)	2(64)
WIK-10	WIKKIWKKIK-NH_2_	1369	8(32)	8(32)	8(64)	2(8)	2(16)
RIW-10	RRIIRRIWRW-NH_2_	1509	8(16)	16(32)	8(16)	2(4)	2(8)
RWI-10	RRIWRRWIRI-NH_2_	1509	2(4)	16(32)	8(16)	1(4)	2(8)
WIR-10	WIRRIWRRIR-NH_2_	1509	2(4)	4(8)	4(8)	1(1)	2(2)
KIW-14	KKIIKKIIKKIWKW-NH_2_	1852	4(4)	2(2)	4(8)	1(2)	0.5(1)
KWI-14	KKIWKKWIKKIIKI-NH_2_	1852	4(4)	2(2)	4(8)	1(2)	0.5(1)
WIK-14	WIKKIWKKIIKKIK-NH_2_	1852	4(4)	2(2)	2((2)	1(1)	0.5(1)
WIKE-14	WIKKIWKKIIKEIK-NH_2_	1853	4(64)	8(32)	16(128)	2(8)	4(8)
Melittin	GIGAVLKVLTTGLPALISWIKRKRQQ-NH_2_	2845	2(2)	2(16)	2(2)	2(2)	1(2)

* Ec: *Escherichia coli*; Pa: *Pseudomonas aeruginosa*; Sa: *Staphylococcus aureus*; Bs: *Bacillus subtilis*; Lm: *Listeria monocytogenes*. All MIC values were measured in the presence of 10 mM sodium phosphate and phosphate-buffered saline (PBS) (numbers in parentheses).

**Table 2:** Maximum tryptophan emission wavelength of the designed peptides with aqueous buffers and membrane environments (lipid vesicles).

Peptide	Aqueous Condition		PE/PG Vesicle (7:3, *w*/*w*) ^c^		PG/CL Vesicle (1:1, *w*/*w*) ^c^		PC/CH/SM Vesicle (1:1:1, *w*/*w*/*w*)	
	Buffer I ^a^	Buffer II ^b^	Buffer I	Buffer II	Buffer I	Buffer II	Buffer II ^c^	Buffer II ^d^
KIW-10	352	356	343	346	340	341	355	351
KWI-10	354	354	338	345	340	341	354	352
WIK-10	354	354	334	339	331	332	354	354
RIW-10	352	356	339	341	339	339	356	353
RWI-10	353	355	338	338	337	338	355	353
WIR-10	352	355	337	337	337	338	354	354
KIW-14	353	357	340	340	339	340	347	343
KWI-14	353	357	332	332	331	332	349	352
WIK-14	356	356	318	318	321	321	354	350
WIKE-14	353	356	332	337	322	328	353	352

^a,b^ The buffers were 10 mM HEPES (pH 7.4) with 10 mM NaCl (buffer I) or 150 mM NaCl and 5 mM MgCl_2_ (buffer II), respectively. ^c,d^ Peptide/lipid molar ratio is 1:100, except for d, which is 1:20.

## Data Availability

Not applicable.
